# CRISPR/Cas9-mediated nexilin deficiency interferes with cardiac contractile function in zebrafish in vivo

**DOI:** 10.1038/s41598-023-50065-9

**Published:** 2023-12-19

**Authors:** Janessa Hofeichner, Bernd Martin Gahr, Magdalena Huber, Alena Boos, Wolfgang Rottbauer, Steffen Just

**Affiliations:** 1 Molecular Cardiology, Department of Internal Medicine II, Ulm University, Ulm, Germany; 2 Department of Internal Medicine II, Ulm University, Ulm, Germany

**Keywords:** Developmental biology, Molecular biology, Physiology, Cardiology, Diseases

## Abstract

Nexilin (NEXN) plays a crucial role in stabilizing the sarcomeric Z-disk of striated muscle fibers and, when mutated, leads to dilated cardiomyopathy in humans. Due to its early neonatal lethality in mice, the detailed impact of the constitutive homozygous *NEXN* knockout on heart and skeletal muscle morphology and function is insufficiently investigated. Here, we characterized a constitutive homozygous CRISPR/Cas9-mediated *nexn* knockout zebrafish model. We found that Nexn deficient embryos developed significantly reduced cardiac contractility and under stressed conditions also impaired skeletal muscle organization whereas skeletal muscle function seemed not to be affected. Remarkably, in contrast to *nexn* morphants, CRISPR/Cas9 *nexn*^*−/−*^ knockout embryos showed a milder phenotype without the development of a pronounced pericardial edema or blood congestion. *nexn*-specific expression analysis as well as whole transcriptome profiling suggest some degree of compensatory mechanisms. Transcripts of numerous essential sarcomeric proteins were massively induced and may mediate a sarcomere stabilizing function in *nexn*^*−/−*^ knockout embryos. Our findings demonstrate the successful generation and characterization of a constitutive homozygous *nexn* knockout line enabling the detailed investigation of the role of *nexn* on heart and skeletal muscle development and function as well as to assess putative compensatory mechanisms induced by the loss of Nexn.

## Introduction

According to the World Health Organization (WHO), cardiovascular disease is the leading cause of death worldwide, accounting for approximately 17.9 million deaths per year^1^. One of the main causes of heart failure is dilated cardiomyopathy (DCM), which leads to the dilation of the heart muscle and thus to impaired contractile function that can cause heart failure and sudden cardiac death^2,3^. DCMs are frequently caused by mutations in genes encoding for proteins such as Titin, α-Actin, Myosin Heavy Chain or Nexilin (NEXN) that make up the sarcomere, the basic contractile unit of striated muscle^2,4,5^.

*NEXN* was first identified in a screen for cardiac proteins associated with DCM in humans^6^. As a F-actin-binding protein, NEXN appears to play a role in linking the actin cytoskeleton to the plasma membrane and is predominantly expressed in cardiomyocytes and skeletal muscle cells^7^. We have shown that Morpholino (MO)-induced knockdown of *nexn* disrupts sarcomeric integrity and Z-disc architecture leading to DCM in zebrafish. Similar structural Z-disk alterations were observed in patients carrying *NEXN* mutations^6^. Furthermore, NEXN deficiency in mice was shown to be associated with the development of DCM^8,9^, endomyocardial fibroelastosis, characterized by increased deposition of collagen and elastin, as well as the intrauterine or early postnatal lethality^6,10^. Recent studies suggest that NEXN is also involved in the initiation and formation of T-tubules, which are required for Ca^2+^ signaling from the sarcoplasmic reticulum in cardiomyocytes^11^. Interestingly, although *NEXN* is also highly expressed in skeletal muscle, loss of NEXN appears to have more deleterious effects on cardiac muscle^6^. Patients with *NEXN* mutations suffer primarily from cardiac dysfunction and do not show skeletal muscle impairments. In zebrafish, defective skeletal muscle structure and function were also found only after a drastic increase in workload^6^.

In the study presented here, we established and characterized a CRISPR/Cas9-mediated homozygous zebrafish model of constitutive Nexn deficiency. The ability of zebrafish to survive up to 5 days post fertilization without a functioning cardiovascular system allows the detailed analysis of the impact of loss of the important sarcomeric protein Nexn. We found that Nexn*-*deficient embryos develop a progressive form of DCM even under basal conditions and additional skeletal muscle defects under stress conditions. Interestingly, the muscular defects observed here are milder than those seen after MO-mediated *nexn* knockdown. Analysis of transcriptional profiles suggests that there are certain compensatory transcriptional changes in CRISPR/Cas9-mediated Nexn-deficient zebrafish embryos, namely the induction of expression of essential sarcomeric proteins, which may mitigate the detrimental loss of Nexn. This zebrafish line will allow to study the role of *nexn* in cardiac and skeletal muscle development and function in more detail and the possible compensatory mechanisms triggered by mutation of *nexn.*

## Results

### CRISPR/Cas9-induced homozygous knockout of *nexn* causes progressive cardiac dysfunction without affecting skeletal muscle function in zebrafish

**Figure 1:**
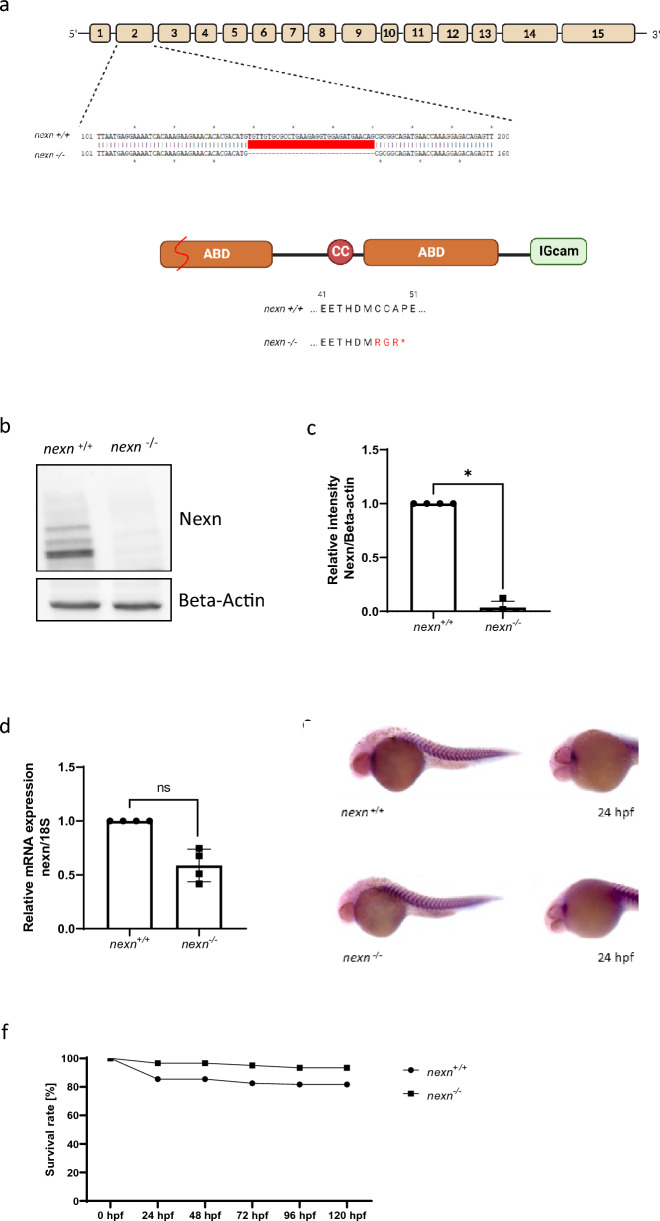
Generation of zebrafish *nexn* knockout by CRISPR/Cas9 gene editing. (**a**) Structure of the *nexn* gene and protein. Nexn consists of a central coiled coil domain (CC) flanked by two actin-binding domains (ABD) and a C-terminal immunoglobulin superfamily class (IGcam). The deletion of 32 nucleotides in exon 2 by CRISPR/Cas9 editing leads to a frameshift and premature stop codon, and thereby to a termination of the Nexn translation after 49 amino acids within the first actin-binding domain. (**b**,**c**) Immunoblot and quantification of protein lysates of *nexn*^*−/−*^ embryos showing reduced Nexn protein levels compared to *nexn*^+*/*+^ embryos at 72 hpf (N = 4, mean ± SD, p < 0.0286 using Wilcoxon test); original blot shown in Supplementary Fig. S1. (**d**) Quantitative real-time PCR of *nexn*^*−/−*^ embryos showing similar *nexn* mRNA levels compared to *nexn*^+*/*+^ embryos at 72 hpf (N = 4, mean ± SD, p = 0.149 using Wilcoxon test). (**e**) in situ hybridization shows *nexn* expression in heart and skeletal muscle at a similar level in *nexn*^*−/−*^ and *nexn*^+*/*+^ embryos at 24 hpf (N = 3, n = 15). (**f**) *nexn*^+*/*+^ and *nexn*^*−/−*^ embryos do not show differences regarding survival rate at any developmental stage (N = 3 with a total number of 87 and 62 embryos, respectively, mean). *ns* not significant, *p < 0.05.

Several studies in different animal models and cardiomyopathy patients have shown that loss of *NEXN* is leading to DCM which is characterized by an impaired contractility of the heart^6,10,12^. Homozygous loss of *NEXN* mostly causes intrauterine or early postnatal lethality^12,13^, impeding the assessment of effects on early muscle development as well as cardiac and skeletal muscle function. In addition to previous studies demonstrating the effects of MO-mediated knockdown of *nexn* in zebrafish, we here generated a constitutive homozygous CRISPR/Cas9-induced *nexn* knockout zebrafish line. A 32 nucleotide deletion within exon 2 leads to the establishment of a premature stop codon causing the termination of protein translation at position 49 within the first actin-binding-domain of Nexn (Fig. 1a). Nexn loss was confirmed via immunoblot analysis where we found a significant reduction of Nexn protein levels in homozygous *nexn*^*−/−*^ embryos (N = 4, p = 0.0286) (Fig. 1b,c and Supplementary Fig. S1). Nevertheless, quantitative RT-PCR (qPCR) analysis revealed that *nexn* mRNA levels were unaltered in *nexn*^*−/−*^ embryos (N = 4, p = 0.1429) (Fig. 1d). Additionally, RNA in situ hybridization showed similar *nexn* expression patterns and quantity in skeletal muscle and heart in both, *nexn*^+*/*+^ and *nexn*^*−/−*^ embryos at 24 h post fertilization (hpf) (Fig. 1e). Furthermore, the Kaplan–Meier curve did not show increased mortality of *nexn*^*−/−*^ compared to *nexn*^+*/*+^ embryos between 24 and 120 hpf (Fig. 1f). In fact, *nexn*^*−/−*^ fish survive to adulthood, are fertile and could be kept for several years. Nevertheless, adult fish showed a tendency towards increased heart weight/body weight ratio in *nexn*^*−/−*^ compared to *nexn*^+*/*+^ fish (N = 3, p = 0.1000) (Supplementary Fig. S2), suggesting a putative late-onset of cardiomyopathy.

**Figure 2:**
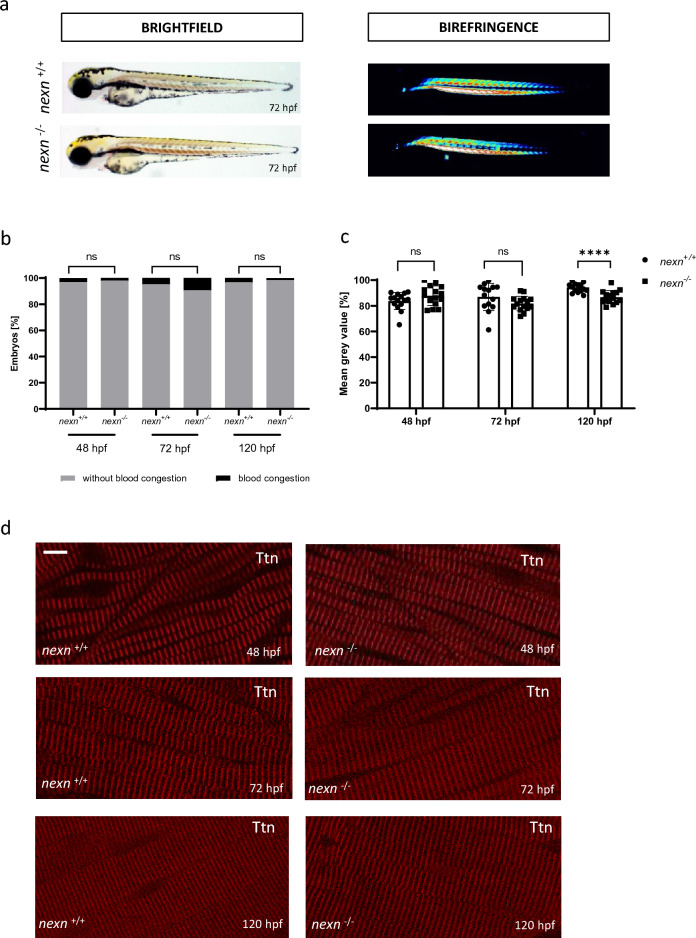
*nexn* knockout does not lead to severe skeletal muscle deficits. (**a**) Brightfield and birefringence images do not reveal phenotypical differences between *nexn*^+*/*+^ and *nexn*^*−/−*^ embryos at 72 hpf. (**b**) Percentage of embryos showing phenotypical abnormalities does not differ between *nexn*^+*/*+^ and *nexn*^*−/−*^ embryos at 48, 72 or 120 hpf (N = 3, mean ± SD, 48 hpf: p > 0.9999, 72 hpf: p = 0.6000, 120 hpf: p > 0.9999 using Mann–Whitney test). (**c**) Densitometric quantification of birefringence signals of *nexn*^*−/−*^ and *nexn*^+*/*+^ embryos reveals significant differences at 120 (N = 3, n = 15, mean ± SD, p < 0.0001 using two-tailed *t*-test) but not 48 or 72 hpf (N = 3, n = 14/15, mean ± SD, 48 hpf: p = 0.1301, 72 hpf: p = 0.1201 using two-tailed *t*-test). (**d**) Immunostaining of *nexn*^+*/*+^ and *nexn*^*−/−*^ embryos at 48, 72 and 120 hpf against Titin does not show muscle disruption. Scale bar (1 µm) refers to all images. *ns* not significant, ****p < 0.0001. Exact values (mean ± SD) are shown in Supplementary Table 1.

**Figure 3:**
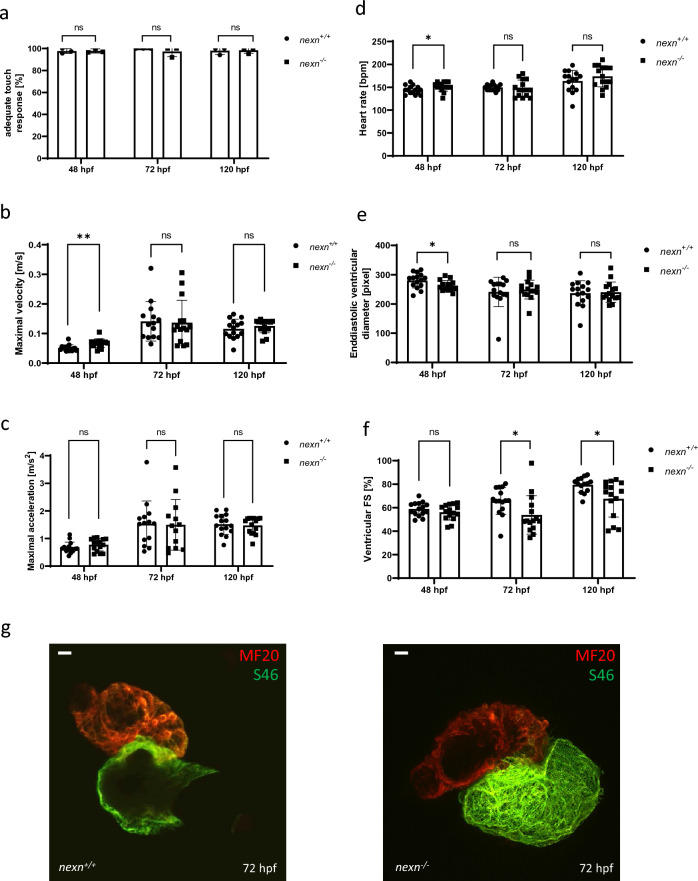
*nexn* knockout causes reduced cardiac function in zebrafish. (**a**) *nexn*^+*/*+^ and *nexn*^*−/−*^ embryos do not show differences regarding responsiveness to mechanical stimuli at any developmental stage (N = 3, mean ± SD, p > 0.9999 for all using Mann–Whitney test). (**b**) *nexn*^*−/−*^ embryos showing increased velocity at 48 hpf (N = 3, n = 15, mean ± SD, p = 0.0017 using two-tailed *t*-test) but not at later time points (N = 3, n = 14/15, mean ± SD, 72 hpf: p = 0.8709, 120 hpf: p = 0.3610 using two-tailed *t*-test). (**c**) Acceleration is at a similar level in *nexn*^+*/*+^ and *nexn*^*−/−*^ embryos at 48, 72 and 120 hpf (N = 3, n = 14/15, mean ± SD, 48 hpf: p = 0.2121, 72 hpf: p = 0.9312, 120 hpf: p = 0.7324 using two-tailed *t*-test). (**d**) Heart rate was increased in *nexn*^*−/−*^ embryos at 48 hpf (N = 3, n = 15, mean ± SD, p = 0.0404 using two-tailed *t*-test) but at a similar level as in *nexn*^+*/*+^ embryos at 72 and 120 hpf (N = 3, n = 15, mean ± SD, 72 hpf: p = 0.6927, p = 0.2300 using two-tailed *t*-test). (**e**) Whereas enddiastolic ventricular diameter was significantly decreased in *nexn*^*−/−*^ embryos at 48 hpf (N = 3, n = 15, mean ± SD, p = 0.0350 using two-tailed *t*-test), it was not affected at 72 or 120 hpf (N = 3, n = 15, mean ± SD, 72 hpf: p = 0.6099, 120 hpf: p = 0.8075 using two-tailed *t*-test). (**f**) Analysis of ventricular fractional shortening does not show altered heart contractility in *nexn*^*−/−*^ at 48 hpf (N = 3, n = 15, mean ± SD, p = 0.2315 using two-tailed *t*-test) but reduced contractility at 72 and 120 hpf (N = 3, n = 15, mean ± SD, 72 hpf: p = 0.0300, 120 hpf: p = 0.0141 using two-tailed *t*-test). (**g**) MF20/S46 staining shows properly differentiated ventricle and atrium in *nexn*^*−/−*^ and *nexn*^+*/*+^ embryos at 72 hpf. Scale bar (20 µM) refers to both images. *FS* fractional shortening, *ns* not significant, *p < 0.05, **p < 0.01. Exact values (mean ± SD) are shown in Supplementary Table 1.

Interestingly, in contrast to the MO-mediated knockdown of *nexn* which led to the development of a pronounced pericardial edema, reduced ventricular fractional shortening (FS) and impaired skeletal muscle organization (Supplementary Fig. S3), overall morphology of *nexn*^+*/*+^ and *nexn*^*−/−*^ embryos did not differ significantly (Fig. 2a,b and Supplementary Fig. S4). To assess skeletal muscle function, birefringence analysis was performed. Signal intensity was at the same level in *nexn*^*−/−*^ and *nexn*^+*/*+^ embryos at 48 and 72 hpf (48 hpf: N = 3, n = 15, p = 0.1301); (72 hpf: N = 3, n = 14/15, p = 0.1201) but significantly reduced in *nexn*^*−/−*^ embryos at 120 hpf (N = 3, n = 15, p < 0.0001) (Fig. 2a,c). Nevertheless, muscle structure of *nexn*^*−/−*^ embryos, assessed by the immunostaining of the sarcomeric protein Titin, was indistinguishable from *nexn*^+*/*+^ embryos at 48, 72 and 120 hpf (N = 3, n = 15) (Fig. 2d). Also responsiveness to mechanical stimuli seemed not to be affected at any stage (N = 3, p > 0.9999 for all) (Fig. 3a). For detailed analysis of the touch-evoked flight response, we calculated the velocity and acceleration of *nexn*^+*/*+^ and *nexn*^*−/−*^ embryos. Movement is illustrated representative for 48, 72 and 120 hpf (Supplementary Fig. S5). Velocity appeared increased in *nexn*^*−/−*^ compared to *nexn*^+*/*+^ embryos at 48 hpf (N = 3, n = 15, p = 0.0017) but not at later time points (72 hpf: N = 3, n = 14/15, p = 0.8709); (120 hpf N = 3, n = 15, p = 0.3610). Acceleration was unaffected in *nexn*^*−/−*^ compared to *nexn*^+*/*+^ embryos at all analyzed stages (48 hpf: N = 3, n = 15, p = 0.2121); (72 hpf: N = 3, n = 14/13, p = 0.8709); (120 hpf N = 3, n = 15, p = 0.3610) (Fig. 3b,c). To analyze heart function in *nexn*^*−/−*^ embryos, heart rate, enddiastolic ventricular diameter (EVD) and FS were assessed at 48, 72 and 120 hpf. Heart rate was significantly increased in *nexn*^*−/−*^ embryos at 48 hpf (N = 3, n = 15, p = 0.0404) but did not differ at later developmental stages (72 hpf: N = 3, n = 15, p = 0.6927); (120 hpf: N = 3, n = 15, p = 0.2300) (Fig. 3d). EVD was significantly reduced in 48 hpf *nexn*^*−/−*^ embryos (N = 3, n = 15, p = 0.0350) but at similar levels as in *nexn*^+*/*+^ embryos at 72 and 120 hpf (72 hpf: N = 3, n = 15, p = 0.6099); (120 hpf: N = 3, n = 15, p = 8075) (Fig. 3e). FS was not significantly affected at 48 hpf (N = 3, n = 15, p = 0.2315) but significantly decreased at 72 as well as 120 hpf in *nexn*^*−/−*^ compared to *nexn*^+*/*+^ embryos (72 hpf: N = 3, n = 15, p = 0.0300); (120 hpf: N = 3, n = 14/15, p = 0.0141) (Fig. 3f). Immunostaining against Meromyosin (MF20, red) and atrial Myosin Heavy Chain (S46, green) showed proper cardiac differentiation and specification into ventricle and atrium in *nexn*^*−/−*^ embryos (N = 3, n = 15) (Fig. 3g). Taken together, these findings are in line with already published results of Nexn loss leading to cardiac dysfunction without significantly interfering with skeletal muscle structure and function^6^.

### Increased workload results in impaired skeletal muscle function in Nexn deficient zebrafish embryos

**Figure 4:**
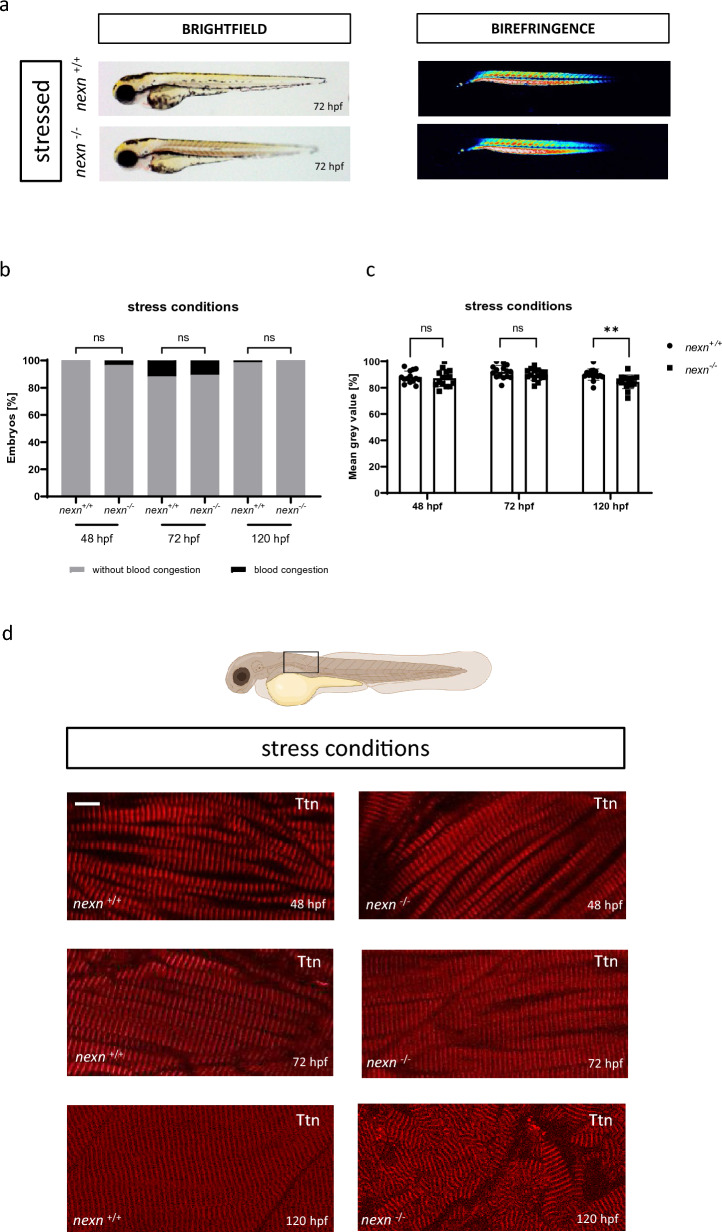
Increased muscular workload causes skeletal muscle disruption in later developmental stages of *nexn* knockout embryos. (**a**) Brightfield and birefringence images do not reveal phenotypical differences between *nexn*^+*/*+^ and *nexn*^*−/−*^ embryos at 72 hpf. (**b**) Percentage of embryos showing phenotypical abnormalities does not differ between *nexn*^+*/*+^ and *nexn*^*−/−*^ embryos at 48, 72 or 120 hpf (N = 3, mean ± SD, 48 hpf: p > 0.9999, 72 hpf: p = 0.8000, 120 hpf: p > 0.9999 using Mann–Whitney test). (**c**) Densitometric quantification of birefringence signals of *nexn*^*−/−*^ and *nexn*^+*/*+^ embryos after stress reveals significant differences at 120 (N = 3, n = 15, mean ± SD, p < 0.0046 using two-tailed *t*-test) but not 48 or 72 hpf (N = 3, n = 14/15, mean ± SD, 48 hpf: p = 0.6516, 72 hpf: p = 0.2193 using two-tailed *t*-test). (**d**) Immunostaining of *nexn*^+*/*+^ and *nexn*^*−/−*^ embryos after stress against Titin does not show altered muscle fibers at 48 and 72 hpf but severe muscle disruption in cranial regions (location shown in schematic illustration; created with biorender.com) at 120 hpf. Scale bar (1 µm) refers to all images. *ns* not significant, **p < 0.01. Exact values (mean ± SD) are shown in Supplementary Table 1.

**Figure 5:**
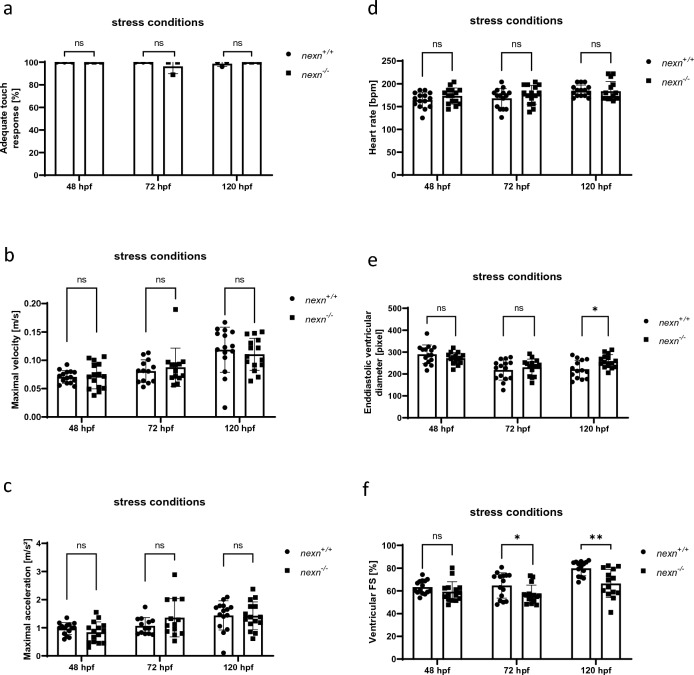
Increased muscular workload does not lead to aggravated cardiac dysfunction in *nexn* knockout zebrafish. (**a**) *nexn*^+*/*+^ and *nexn*^*−/−*^ embryos do not show significant differences regarding responsiveness to mechanical stimuli at 48, 72 or 120 hpf (N = 3, mean ± SD, p > 0.9999 for all using Mann–Whitney test). (**b**) Maximal velocity was not affected at any developmental stage after increasing workload (N = 3, n = 15, mean ± SD, 48 hpf: p = 0.7875, 72 hpf: p = 0.5428, 120 hpf: p = 0.5202 using two-tailed *t*-test). (**c**) Maximal acceleration is at a similar level in *nexn*^+*/*+^ and *nexn*^*−/−*^ embryos at 48, 72 and 120 hpf (N = 3, n = 15, mean ± SD, 48 hpf: p = 0.2460, 72 hpf: p = 0.1754, 120 hpf: p = 0.9284 using two-tailed *t*-test). (**d**) Heart rate of *nexn*^*−/−*^ embryos was at a similar level as in *nexn*^+*/*+^ embryos at 48, 72 and 120 hpf (N = 3, n = 15, mean ± SD, 48 hpf: p = 0.1738, 72 hpf: p = 0.3121, 120 hpf: p = 0.8520 using two-tailed *t*-test). (**e**) Increased workload does not affect enddiastolic ventricular diameter at 48 and 72 hpf (N = 3, n = 15, mean ± SD, 48 hpf: p = 0.1807, 72 hpf: p = 0.4152 using two-tailed *t*-test) but leads to increased enddiastolic diameter at 120 hpf (N = 3, n = 15, mean ± SD, p = 0.0017 using two-tailed *t*-test). (**f**) Analysis of ventricular fractional shortening does not show altered heart contractility in *nexn*^*−/−*^ compared to *nexn*^+*/*+^ embryos at 48 hpf (N = 3, n = 15, mean ± SD, p = 0.1538 using two-tailed *t*-test) but reduced fractional shortening at 72 and 120 hpf (N = 3, n = 15, mean ± SD, 72 hpf: p = 0.0379, 120 hpf: p = 0.0020 using two-tailed *t*-test). *FS* fractional shortening, *ns* not significant, *p < 0.05, **p < 0.01. Exact values (mean ± SD) are shown in Supplementary Table 1.

In *nexn* morphant embryos, it was shown that skeletal muscle dysfunction can be provoked by increasing their workload via electrical stimulation^6^. Here, we aimed to mimic this approach by increasing the viscosity of the medium by adding 1% methylcellulose and incubating embryos for 2 h^14,15^. Stressing the embryos did not lead to morphological alterations of *nexn*^*−/−*^ embryos compared to *nexn*^+*/*+^ at 48, 72 or 120 hpf (Fig. 4a,b and Supplementary Fig. S4). Similar to the unstressed embryos, skeletal muscle organization and function was tested. Birefringence analysis did not reveal altered signal intensity in *nexn*^*−/−*^ embryos at 48 and 72 hpf (48 hpf: N = 3; n = 14/15, p = 0.6516); (72 hpf: N = 3, n = 15, p = 0.2193). Again, 120 hpf *nexn*^*−/−*^embryos showed a significant reduction in birefringence signal, also under stress conditions (N = 3, n = 15, p = 0.0046) (Fig. 4c). Immunostaining of Titin showed organized myofibrils at 48 and 72 hpf *nexn*^*−/−*^ embryos after methylcellulose stress. Surprisingly, 120 hpf *nexn*^*−/−*^ embryos seemed to have unaltered myofibrillar assembling in caudal areas (data now shown) of their skeletal muscles, whereas they showed unorganized myofibrils in more cranial regions (exact location shown in schematic illustration) (Fig. 4d). Touch-evoked flight response was unaltered in *nexn*^*−/−*^ compared to *nexn*^+*/*+^ embryos independently of their developmental stage (48 hpf: N = 3, p > 0.9999 for all) (Fig. 5a). A more detailed look at the motility of the embryos under stress conditions revealed no significant changes regarding velocity (48 hpf: N = 3, n = 15, p = 0.7875); (72 hpf: N = 3, n = 13, p = 0.5478); (120 hpf: N = 3, n = 15, p = 0.5202) or acceleration (48 hpf: N = 3, n = 15, p = 0.2460); (72 hpf: N = 3, n = 13, p = 0.1754); (120 hpf: N = 3, n = 15, p = 0.9284) in 48, 72 or 120 hpf *nexn*^*−/−*^ embryos. (Fig. 5b,c). Movement of *nexn*^+*/*+^ and *nexn*^*−/−*^ embryos is illustrated representatively (Supplementary Fig. S5). Moreover, cardiac function under stress conditions was assessed. Heart rate of *nexn*^*−/−*^ embryos was at a similar level when compared to *nexn*^+*/*+^ embryos independently of their developmental stage (48 hpf: N = 3, n = 15, p = 0.1738); (72 hpf: N = 3, n = 15, p = 0.3121); (120 hpf: N = 3, n = 15, p = 0.8520) (Fig. 5d). Whereas EVD was not affected at 48 and 72 hpf (48 hpf: N = 3, n = 15, p = 0.1807); (72 hpf: N = 3, n = 15, p = 0.4152), 120 hpf *nexn*^*−/−*^ embryos had a significantly increased EVD (N = 3, n = 14/15, p = 0.0117) (Fig. 5e). Similar to unstressed conditions, stressed *nexn*^*−/−*^ embryos developed a significant reduction of FS starting from 72 hpf (72 hpf: N = 3, n = 15, p = 0.0379); (120 hpf: N = 3, n = 14/15, p = 0.0020) whereas FS was unaltered at 48 hpf (N = 3, n = 15, p = 0.1807) (Fig. 5f). These effects are in line with previous studies showing myofibrillar disruption in skeletal muscle tissue under stress conditions^6^.

### Isoproterenol-induced heart rate increase does not cause aggravating symptoms in Nexn deficient zebrafish embryos

**Figure 6:**
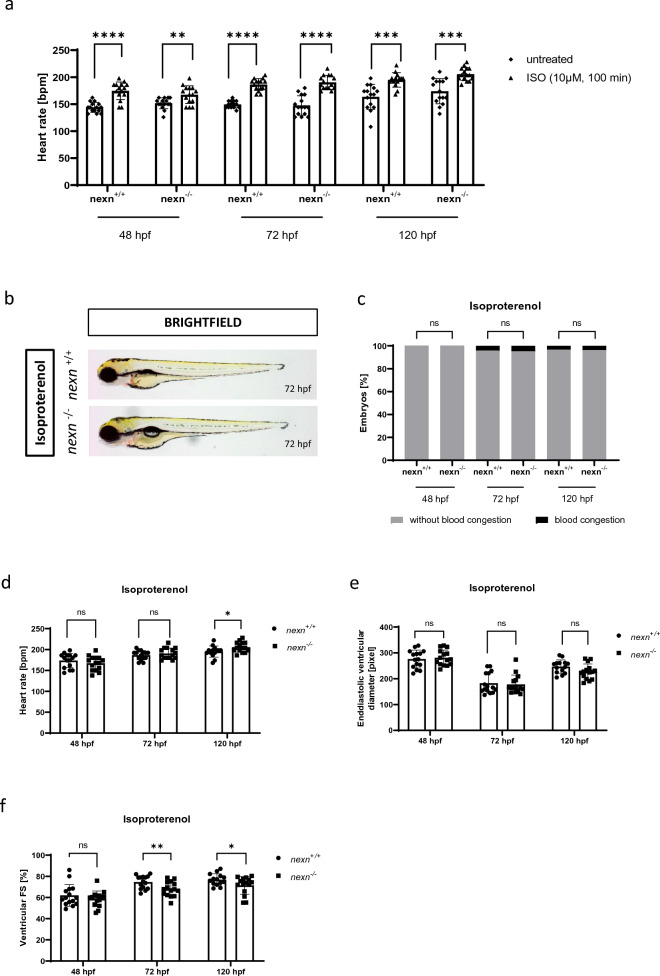
Increasing heart rate does not cause severe changes in cardiac functionality. (**a**) Isoproterenol treatment increases heart rate in *nexn*^+*/*+^ and *nexn*^*−/−*^ embryos at all developmental stages (N = 3, n = 14/15, mean ± SD, 48 hpf *nexn*^+*/*+^: p < 0.0001, 48 hpf *nexn*^*−/−*^: p = 0.0085, 72 hpf *nexn*^+*/*+^: p < 0.0001, 72 hpf *nexn*^*−/−*^: p < 0.0001, 120 hpf *nexn*^+*/*+^: p = 0.0002, 120 hpf *nexn*^*−/−*^: p = 0.0001 using two-tailed *t*-test). (**b**) Brightfield images do not reveal phenotypical differences between *nexn*^+*/*+^ and *nexn*^*−/−*^ embryos at 72 hpf. (**c**) Percentage of embryos showing phenotypical abnormalities does not differ between *nexn*^+*/*+^ and *nexn*^*−/−*^ embryos at 48, 72 or 120 hpf (N = 3, mean ± SD, p > 0.9999 for all using Mann–Whitney test). (**d**) Heart rate of *nexn*^*−/−*^ embryos is at a similar level as in *nexn*^+*/*+^ embryos at 48, and 72 hpf but increased at 120 hpf (N = 3, n = 15, mean ± SD, 48 hpf: p = 0.3112, 72 hpf: p = 0.3690, 120 hpf: p = 0.0350 using two-tailed *t*-test). (**e**) Increased heart rate does not affect enddiastolic ventricular diameter at 48, 72 and 120 hpf (N = 3, n = 15, mean ± SD, 48 hpf: p > 0.9999, 72 hpf: p = 0.7588, 120 hpf: p = 0.0701 using two-tailed *t*-test). (**f**) Analysis of ventricular fractional shortening does not show altered heart contractility in *nexn*^*−/−*^ compared to *nexn*^+*/*+^ embryos at 48 hpf (N = 3, n = 15, mean ± SD, p = 0.3284 using two-tailed *t*-test) but reduced fractional shortening at 72 and 120 hpf (N = 3, n = 15, mean ± SD, 72 hpf: p = 0.0099, 120 hpf: p = 0.0498 using two-tailed *t*-test). *FS* fractional shortening, *ns* not significant, *p < 0.05, **p < 0.01. Exact values (mean ± SD) are shown in Supplementary Table 1.

As we observed an increased heart rate in unstressed *nexn*^*−/−*^ compared to unstressed *nexn*^+*/*+^ embryos at 48 hpf but not at later time points or under stressed conditions, we were interested if some of the observed effects may be dependent on elevated heart rate (and thus higher workload) at certain stages of development. For increasing heart rate, zebrafish were treated with Isoproterenol^16^ (N = 3, n = 15 for all; (48 hpf *nexn*^+*/*+^: p < 0.0001); (48 hpf *nexn*^*−/−*^: p = 0.0085); (72 hpf *nexn*^+*/*+^: p < 0.0001); (72 hpf *nexn*^*−/−*^: p < 0.0001); (120 hpf *nexn*^+*/*+^: p = 0.0002); (120 hpf *nexn*^*−/−*^: p = 0.0001) (Fig. 6a). Isoproterenol-induced increase of heart rate did not lead to morphological changes at any developmental stage (Fig. 6b,c and Supplementary Fig. S4). A difference in heart rate between *nexn*^+*/*+^ and *nexn*^*−/−*^ embryos could not be observed at 48 and 72 hpf (48 hpf: N = 3, n = 15, p = 0.3112); (72 hpf: N = 3, n = 15, p = 0.3690) but heart rate was slightly increased in *nexn*^*−/−*^ embryos at 120 hpf (N = 3, n = 14/15, p = 0.0350) (Fig. 6d). EVD was not affected at any developmental stage (48 hpf: N = 3, n = 15, p > 0.9999); (72 hpf: N = 3, n = 15, p = 0.7580); (120 hpf: N = 3, n = 14/15, p = 0.0701) (Fig. 6e). Similar to unstressed *nexn*^*−/−*^ embryos and *nexn*^*−/−*^ embryos being stressed with methylcellulose, also *nexn*^*−/−*^ embryos being treated with Isoproterenol did not show reduced FS compared to *nexn*^+*/*+^ embryos at 48 hpf (N = 3, n = 15, p = 0.3284). FS was significantly reduced starting from 72 hpf (72 hpf: N = 3, n = 15, p = 0.0099); (120 hpf: N = 3, n = 14/15, p = 0.0498) (Fig. 6f). Taken together, increasing heart rate by Isoproterenol did augment cardiac contractile dysfunction in Nexn deficient embryos.

**Figure 7:**
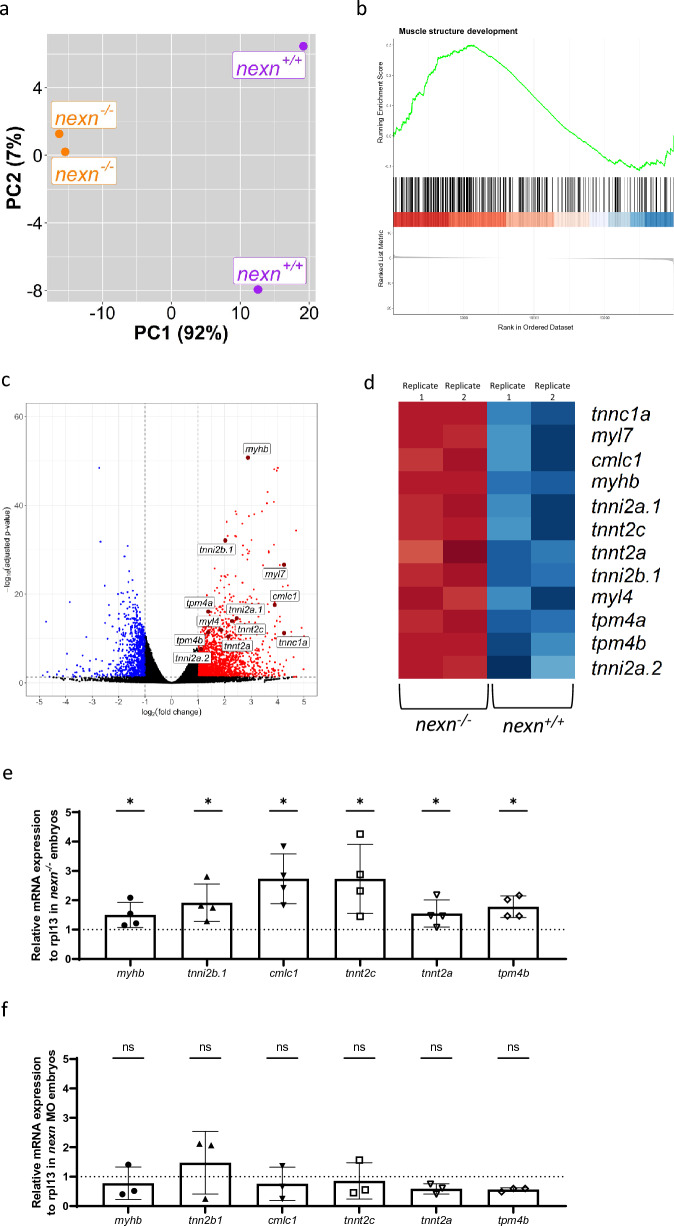
Nexn deficiency leads to upregulation of several genes encoding for sarcomeric proteins. (**a**) Principal component analysis (PCA) plot showing *nexn*^+*/*+^ and *nexn*^*−/−*^ embryos (72 hpf) being clearly separated by PC1 (N = 2, n = 25). (**b**) Gene set enrichment analysis of the Gene Ontology term “muscle structure development” showing an overrepresentation of upregulated genes in *nexn*^*−/−*^ embryos (normalized ES = 1.84; adjusted p = 1.14E−07). (**c**) Volcano plot of differentially expressed genes in *nexn*^*−/−*^ compared to *nexn*^+*/*+^ embryos. Upregulated genes shown in red and downregulated genes shown in blue (adjusted p-value < 0.05 and |log2(FC)| > 0.5). Selected genes are labeled. (**d**) Heatmap showing selected genes of interest with clear difference regarding expression between *nexn*^+*/*+^ and *nexn*^*−/−*^ embryos. High read counts shown in red and low read counts in blue. (**e**) Quantitative real-time PCR showing significantly increased *myhb*, *tnni2b.1*, *cmlc1*, *tnnt2c*, *tnnt2c*, *tnnt2a* and *tpm4b* transcript levels in *nexn*^*−/−*^ compared to *nexn*^+*/*+^ embryos at 72 hpf. (**f**) Quantitative real-time PCR showing unaltered levels of *myhb*, *tnni2b.1*, *cmlc1*, *tnnt2c*, *tnnt2c*, *tnnt2a* and *tpm4b* in *nexn* MO-injected embryos compared to Ctrl MO-injected embryos at 72 hpf. *ns* not significant, *p < 0.05. Exact values (mean ± SD) are shown in Supplementary Table 1.

The fact that the cardiac phenotype in *nexn*^*−/−*^ embryos was not as severe as the phenotype observed in *nexn* morphants, indicates that CRISPR/Cas9-induced *nexn* knockout might trigger compensatory mechanisms. As shown by qPCR and *nexn-*specific in situ hybridization, we found that *nexn* mRNA was not downregulated in *nexn*^*−/−*^ embryos (Fig. 1), implying that the nonsense-mediated mRNA decay (NMD) pathway was not induced by the 32 nucleotide deletion within the *nexn* gene and thereby classical genetic compensation mechanisms, as for example observed in CRISPR-mediated Bag3 mutants^15^, might not be triggered in *nexn* knockout embryos. Nevertheless, to assess whether compensatory transcriptional mechanisms were present in *nexn*^*−/−*^ embryos, we conducted RNA-sequencing analysis. The PCA showed *nexn*^+*/*+^ and *nexn*^*−/−*^ clusters that were clearly separated by the first principal component indicating that variances in the data set were most likely caused by the mutation (Fig. 7a). Using differential expression analysis, we found 2094 genes being significantly up- (log2(FC) > 0.5) and 968 genes being significantly downregulated (log2(FC) < -0.5), (adjusted p < 0.05) in *nexn*^*−/−*^ embryos (Supplementary Table 2). Concordant to our qRT-PCR results (Fig. 1d), *nexn* mRNA levels were also unaltered according to the RNA-sequencing data set (adjusted p = 1.14E−05, log2(FC) = −0.896133743). To identify significantly enriched GO-terms, we performed Gene Set Enrichment Analysis using the biological processed terms of the GO database and found muscle structure development to be activated in *nexn*^*−/−*^ embryos (Supplementary Fig. S5) and showing an overrepresentation of upregulated genes in *nexn*^*−/−*^ embryos (normalized ES = 1.84, adjusted p = 1.14E−07) (Fig. 7b). Having a closer look at this GO-term, we found several genes encoding for sarcomeric proteins being significantly upregulated in *nexn*^*−/−*^ embryos including different members of the Myosin, Tropomyosin and Troponin family which are highlighted in the volcano plot and heat map (Fig. 7c,d). In addition to RNASeq, qPCR analysis confirmed the significant upregulation of *myhb*, *tnni2b.1*, *cmlc1*, *tnnt2c*, *tnnt2a* and *tpm4b* transcript levels in *nexn*^*−/−*^ embryos (N = 4, p = 0.0286 for all) (Fig. 7e). In contrast, transcript levels of those genes were unaltered in *nexn* MO-injected embryos (N = 3; p = 0.7000 for all) (Fig. 7f).

These findings suggest that the compensatory transcriptional induction of the expression of essential sarcomeric proteins might mitigate the detrimental loss of Nexn in *nexn*^*−/−*^ embryos.

## Discussion

In humans, mutations in the *NEXN* gene were described to cause DCM which is characterized by a dilation of the myocardium and therefore an impaired cardiac contractility^6^. Similar pathological cardiac alterations were observed after *Nexn* knockout in mice and the MO-mediated knockdown of *nexn* in zebrafish^6,8,9^. Nevertheless, although the critical function of *NEXN* in the heart appears to be conserved across vertebrate species, its detailed structural or molecular role is still insufficiently understood. Here, we established and characterized for the first time a CRISPR/Cas9-induced homozygous zebrafish model of constitutive Nexn deficiency to assess the detailed role of Nexn loss in the development and function of the heart but also skeletal muscle. We found Nexn deficient embryos to develop a progressive form of DCM without involvement of skeletal muscles under physiological conditions. After increasing workload, also skeletal muscle developed a myopathic phenotype. Remarkably, the observed pathologies in the CRISPR/Cas9 mutants were milder than the defects evoked by the MO-mediated knockdown of *nexn*, suggesting compensatory mechanisms to be induced in *nexn*^*−/−*^ mutants. *nexn* mRNA was not found degraded by the NMD pathway in the CRISPR/Cas9 mutants, likely excluding classical genetic compensation as compensatory route. Nevertheless, by transcriptional profiling, the massive transcriptional activation of important sarcomeric proteins was found, implying their upregulation to contribute to the protection of the sarcomere from Nexn deficiency induced Z-disk destabilization.

In line with already published data of MO-mediated Nexn deficiency in zebrafish, CRISPR/Cas9-induced *nexn*^*−/−*^ knockout embryos showed a reduced cardiac contractility^6^. This effect appeared starting from 72 hpf, suggesting a progressive impairment of the heart muscle. Indeed, it’s also known from patients with *NEXN* mutations that their medical conditions are deteriorating over time^17^. Interestingly, in contrast to one of the main symptoms of DCM, we did not observe an enlarged and dilated ventricle in *nexn*^*−/−*^ embryos under physiological conditions. In detail, at 48 hpf the EVD of Nexn deficient embryos was even smaller compared to *nexn*^+*/*+^ embryos which was accompanied by an increased heart rate. The abolishment of this phenotype at later developmental stages could indicate a progressive dilation of the ventricle. This hypothesis is supported by the finding that *nexn*^*−/−*^ embryos developed an enlarged ventricle at 120 hpf when increasing the workload on the heart. Additionally, this could also be caused by different functions of *nexn* during different stages of development which may provide a basis for further impairments at later stages. Whereas the most studied function of *nexn* is the stabilization of the sarcomeric Z-disk^6^, it was also shown that *nexn* seems to be involved in the formation of cardiac T-tubules. Thus, *nexn* obviously plays a role in Ca^2+^-signaling as T-tubules are critical for the coordinated Ca^2+^-flux from the sarcoplasmic reticulum into cardiomyocytes^11^. Recent studies suggest high serum Ca^2+^ levels being associated with a reduced EVD^18^. In this context, the further investigation of the role of *nexn* in Ca^2+^-signaling would be interesting. Furthermore, it remained unclear if reduced EVD is resulting in an increased heart rate in *nexn*^*−/−*^ embryos or vice versa. It is known that a smaller ventricle is leading to an elevated heart rate to compensate for the reduced ejection fraction^19^. In contrast, also increased heart rates appear to be able to lead to the development of smaller ventricles^20,21^. Interestingly, heart rate reduction in children suffering from DCM seems to be able to improve cardiac contractility and ejection fraction^22^. In this context, we induced tachycardia in *nexn*^+*/*+^ and *nexn*^*−/−*^ embryos by incubating them with Isoproterenol at 48, 72 and 120 hpf. In contrast to literature, an increase of heart rate could already be seen at 48 hpf^16^. At 72 and 120 hpf, Isoproterenol-mediated induction of tachycardia did not lead to any additional negative effects on contractile performance compared to unstressed embryos. Strikingly, after isoproterenol treatment, EVDs of *nexn*^+*/*+^ and *nexn*^*−/−*^ embryos were indistinguishable suggesting that rather reduced EVD in unstressed *nexn*^*−/−*^ embryos was influencing heart rate than vice versa.

Although strongly expressed in skeletal muscle, Nexn deficiency appeared not to have severe effects on skeletal muscle organization, function or motility in unstressed *nexn*^*−/−*^ embryos. As shown in previous studies, we were able to provoke sarcomeric disarray and impaired skeletal muscle function in *nexn*^*−/−*^ embryos by increasing their workload^6^. Interestingly, this effect was exclusively seen in cranial regions but not in more caudal areas of the skeletal muscle of the trunk. The cranial somites are the first ones to form during development and thus are the oldest sarcomeres. Together with the fact that this effect can only be recognized under stressed conditions, it implies that skeletal muscle disorganization may be progressive as observed in other diseases affecting the skeletal muscle such as Duchenne Muscular Dystrophy^23^. Nevertheless, this effect did not seem to be severe enough to impair the motility of the *nexn*^*−/−*^ embryos and also patients, to the best of our knowledge, did not report of skeletal muscle involvement^6^. Whereas *NEXN* is predominantly described to be involved in sarcomeric stabilization and T-Tubule formation in cardiac tissue, the current knowledge about the role of *NEXN* in the skeletal muscle includes a role in the glucose uptake of skeletal muscle cells via regulation of insulin receptor substrate 1-signaling^24^. This could be a hint that *NEXN* is having differing functions in heart and skeletal muscle as it is already described for other Z-disk proteins such as α-actinin-2^25,26^.

It is remarkable that CRISPR/Cas9-generated *nexn* knockout embryos in contrast to MO-induced *nexn* knockdown embryos show a milder cardiac phenotype without a pronounced pericardial edema and blood congestion. This phenomenon has already been observed in previous studies regarding other CRISPR/Cas9-mediated gene knockouts and could be induced by NMD which seems to be not present in MO-induced knockdowns^15,27,28^. Briefly, the degradation of mutated mRNA by NMD triggers the transcriptional adaptation of a gene or several genes to potentially compensate for the protein loss and suppress or attenuate the phenotype, a process called genetic compensation. The lacking NMD-mediated degradation of *nexn* mRNA in the CRISPR/Cas9-mediated *nexn*^*−/−*^ embryos opposes this mechanism. Nevertheless, recent studies report a similar mechanism called nonsense-mediated translational repression. This mechanism for example seems to get activated after mRNA degradation failure or if certain complexes needed for NMD cannot be recruited and does not lead to altered mRNA levels^29,30^. As Nexn is a F-actin binding protein, we would suppose another protein with functional homology being upregulated and at least partially compensating for the loss of Nexn. Indeed, by profiling the transcriptome in CRISPR/Cas9-mediated *nexn*^*−/−*^ embryos, we found several genes encoding for important sarcomeric proteins being upregulated in *nexn* mutant embryos, including members of the Troponin, Tropomyosin and Myosin family which are known to interact with Actin^31^ and may, at least to some extent, take over the function of Nexn by stabilizing the sarcomeric Z-disk. Nevertheless, this hypothesis has to be assessed and needs further detailed elucidation.

In summary, we were able to establish and characterize for the first time a homozygous zebrafish model of constitutive Nexn deficiency, enabling the further investigation of the role of *nexn* on heart and skeletal muscle development and function as well as the possible compensatory mechanisms triggered by mutation of *nexn*.

## Methods

### Zebrafish strains and injection procedures

The present study was performed according to institutional approvals (Regierungspräsidium Tübingen No. 1243 and No. 1530, Tierforschungszentrum (TFZ) Ulm University, No. z.183 and o.183-14) coinciding with EU Directive 2010/63/EU. Experiments were in accordance with ARRIVE guidelines and performed according to relevant guidelines and regulations.

Care and breeding of zebrafish, *Danio rerio*, were conducted as described previously^6^.

The lines were generated using CRISPR/Cas9 technology. 400 ng/µl Cas9 protein (Euphoria GmbH, Germany) were injected together with 130 ng/µl tracrRNA (Eurofins Genomics, Germany) and 70 ng/µl of gene-specific crRNA (Eurofins Genomics, Germany) against *nexn* diluted in 200 mM KCl, resulting in the *Nexn* deficient line *nexn*^*−/−*^. If not stated differently, MO injections were conducted with the TüAB wildtype strain. Sequences of tracrRNA and CRISPR RNA oligonucleotides can be found summarized in Fig. S7 in the Supplementary Materials.

MO (Gene Tools, LLC, Oregon, USA) was directed against a splice site of *nexn*^6^. Standard Control (Std Ctrl) MO was used in the respective MO concentration as negative control. Injections were performed during one-cell stage of zebrafish embryos. MO sequences can be found summarized in Fig. S7 in the Supplementary Materials.

### Assessment of heart weight in adult fish

Adult *nexn*^+*/*+^ and *nexn*^*−/−*^ fish (2 years old) were sacrificed and the heart was extracted. The heart weight was assessed in proportion to the body weight and the heart weight/body weight ratio calculated.

### Birefringence analysis and heart and skeletal muscle functional assessment

Images were taken with a Zeiss Axio Zoom V.16 microscope and movies were recorded with a Leica DM IL LED microscope. Cardiac contractility was analyzed by assessing ventricular FS at 48, 72 and 120 hpf as described previously^32^. Instead of the diameter of the ventricle, the diastolic and systolic lumen were measured. EVD was assessed with ImageJ.

Birefringence analysis was performed as previously described^15^.

To assess the responsiveness to mechanical stimuli, zebrafish embryos were touched with the tip of a needle at 48, 72 and 120 hpf. A straightforward flight response was considered as adequate. For detailed analysis, the velocity and acceleration of the embryos were calculated. Briefly, the embryo was put into the center of a petri dish and touched with the needle. Flight response was recorded with 60 frames per second and the video was analyzed using Tracker Video Analysis and Modeling Tool open-source software (physlets.org, accessed on June 07th 2023).

Stressing the embryos was performed in two different approaches. To increase the workload on the heart and skeletal muscles, embryos at 48, 72 and 120 hpf, respectively were incubated in 1% methylcellulose for 2 h as previously described^15^. In a second approach, to increase the workload on the heart, embryos were treated with 10 µM Isoproterenol (Sigma, USA) for 100 min at the stages mentioned above.

### Immunoblotting

Protein lysates were gained from 72 hpf *nexn*^+*/*+^ and *nexn*^*−/−*^ embryos as previously described^33^. For immunoblot analysis, 10 µg of protein were denatured in 5 × Laemmli buffer and separated on 8–16% Tris–glycine gels (BIO-RAD, Hercules, CA, USA). Blotting was performed on polyvinylidene difluoride (PVDF) membranes using the Trans-Blot Turbo System (BIO-RAD, USA). Membranes were blocked in SuperBlock (TBS) Blocking Buffer (Thermo Scientific, Waltham, MA, USA) for 15 min at room temperature (RT). Afterwards, they were incubated with primary antibody diluted 1:1000 in SuperBlock at 4 °C overnight (ON). After washing, the membranes were incubated with the corresponding secondary antibody conjugated to horseradish peroxidase (dilution 1:2500 in TBST (Tris-buffered saline, 0.05% Tween 20)) and developed using Pierce ECL Western Blotting Substrate (Thermo Scientific, USA) and a luminescent image analyzer (Image Quant Las4000 mini)^33^.

Antibodies used: β-Actin (mouse; #A5441; Sigma, Burlington, MA, USA), Nexn (rabbit; designed by Dieter Fürst lab, Bonn, Germany against epitope position 151-581).

### RNA extraction and quantitative real-time PCR

For RNA extraction, 25 *nexn*^+*/*+^ and *nexn*^*−/−*^ embryos were collected at 72 hpf. Extraction was done with the RNeasy^®^ Mini Kit (Qiagen, Hilden, Germany) according to the provided manual. For cDNA synthesis, Superscript III reverse transcriptase (Life Technologies, Carlsbad, CA, USA) was used to reverse transcribe 1 µg of RNA. Quantitative real-time PCR was performed using SYBR-Green master mix (Roche, Basel, Switzerland) according to standard protocols on a Roche Light Cycler 480 II. Sequences of the used primers can be found in Fig. S7 in the Supplementary Materials.

### RNA sequencing and bioinformatic analysis

For RNA sequencing, pooled RNA of 25 embryos of 72 hpf *nexn*^+*/*+^ or *nexn*^*−/−*^ was used. Embryos were obtained by incrossing adult *nexn*^*+/+*^ and *nexn*^−/−^ fish which are offspring of a heterozygous pair and kept in separate tanks for easier handling. Quality control, library preparation and sequencing were performed by Eurofins Germany (Ebersberg, Germany) using the “INVIEW Transcriptome Discover” pipeline. 35 million 150 bp paired reads were sequenced and mapped to the reference genome GRCz11.

During bioinformatic analysis using R, low read counts were removed from the dataset. Differently expressed genes were calculated with the Bioconductor package DESeq2 and annotated with org. Dr.eg.db and AnnotationDbi^34,35^. The volcano plot was visualized using the package ggplot2^36^. The read counts of selected genes were color-coded in heatmaps using gplots. GSEA was performed using the clusterProfiler package^37^ and biological processes terms of the GO database^38^. Single GO-Terms were further analyzed using the gseaplot2 method of the enrichplot package^39^.

### Immunostaining

For sarcomeric Titin staining, *nexn*^+*/*+^ and *nexn*^*−/−*^ embryos (48, 72 and 120 hpf) were fixed in 4% paraformaldehyde (PFA) for 20 min at RT and washed in 0.5% Triton X-100 in PBS three times for 5 min. After incubation in primary antibody (Titin T11, 1:500 in 0.5% Triton X-100 in PBS; Sigma, USA; #T9030) for 1 h at RT, the embryos were washed three times for 5 min in 0.5% Triton X-100 in PBS. Incubation in secondary antibody (mouse IgG2b, 1:1000 in 0.5% Triton X-100 in PBS; Thermo Scientific, USA; #A-21147 ) was performed for 1 h at RT.

For co-staining of MF20 and S46, *nexn*^+*/*+^ and *nexn*^*−/−*^ embryos (72) were fixed in Dent’s Fixative (20% DMSO in MeOH) ON at RT. After bleaching in Dent’s Bleach (10% H_2_O_2_, 20% DMSO in MeOH) ON at RT, the embryos were rehydrated using a descending MeOH row (75% MeOH, 50% MeOH and 25% MeOH in PBT (PBS + 0.1% Tween)) for 20 min each at RT. After washing 3 times for 2 min in PBDT (1% DMSO, 0.1% Tween in PBS), embryos were blocked using 10% fetal bovine serum (FBS) in PBDT for 1.5 h at 4 °C. Incubation in MF20 antibody (1:10 in 1.5% FBS in PBDT; MF20 concentrate, Developmental Studies Hybridoma Bank, Iowa City, IA, USA) was performed ON at 4 °C. After washing the embryos 4 times for 30 min in 1.5% FBS in PBDT, they were incubated in S46 antibody under the same conditions (S46 concentrate, Developmental Studies Hybridoma Bank, Iowa City, IA, USA). Incubation in the corresponding secondary antibodies (goat anti-mouse IgG1 Alexa Fluor 488 and Alexa 555 goat anti-mouse IgG2b (both Invitrogen, Waltham, MA, USA; #A-21121 and  #A-21147)) was performed in 1.5% FBS in PBDT for 3 h at RT.

Images were acquired using a Leica DMi8 microscope (Leica Mikrosysteme Vertrieb GmbH, Wetzlar, Germany).

### In situ hybridization

In situ hybridization was performed essentially according to previous publications using a full-length digoxigenin-labeled *nexn* antisense probe^40^.

### Statistical analysis

Statistical analysis was performed using GraphPad Prism9. qRT-PCR results were analyzed on the mean ΔCT-values using Wilcoxon matched-pairs signed-rank test. The same test was used for Western Blot analysis. All other statistical analyses were performed either using Mann–Whitney test or *t*-test as stated in the corresponding figure legend with statistical significance at p < 0.05. All results are expressed as mean ± standard (SD) deviation.

### Supplementary Information

Supplementary Figures.

Supplementary Table 1.

Supplementary Table 2.

## Data Availability

The dataset generated and analyzed during the current study is available in the Gene Expression Omnibus (GEO) repository (accession number GSE239788).
