# Supplementary material for: CRISPR/Cas9-mediated nexilin deficiency interferes with cardiac contractile function in zebrafish in vivo
Source: Sci Rep. 2023 Dec 19;13:22679. doi: 10.1038/s41598-023-50065-9 (PMC10730861; doi:10.1038/s41598-023-50065-9)
Supplement: Supplementary file 1 — Supplementary Figures. [file 41598_2023_50065_MOESM1_ESM.pdf]

Supplementary Material

Supplementary Figure S1

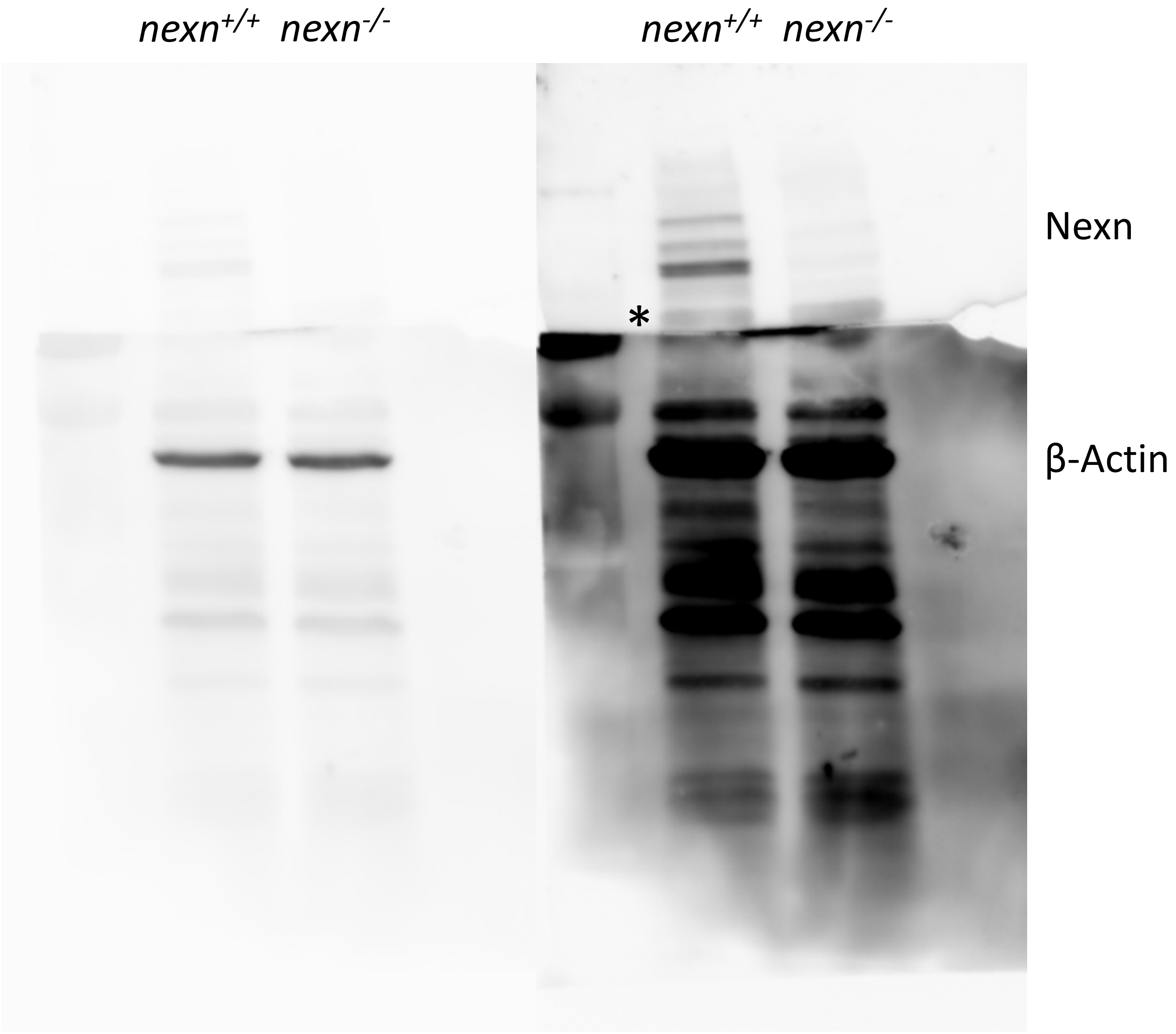

* unspecific band

Supplementary Figure S2

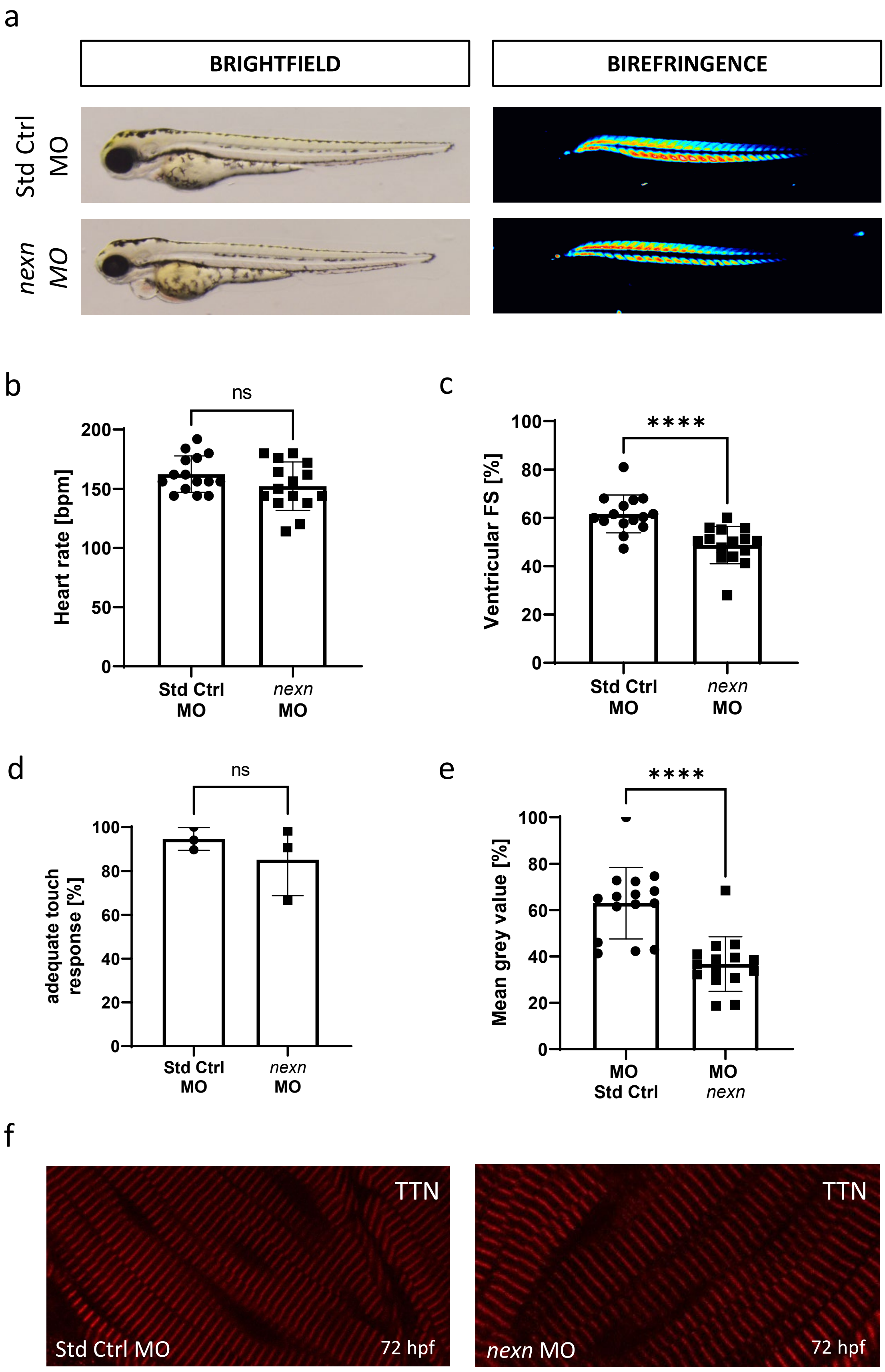

Supplementary Figure S3

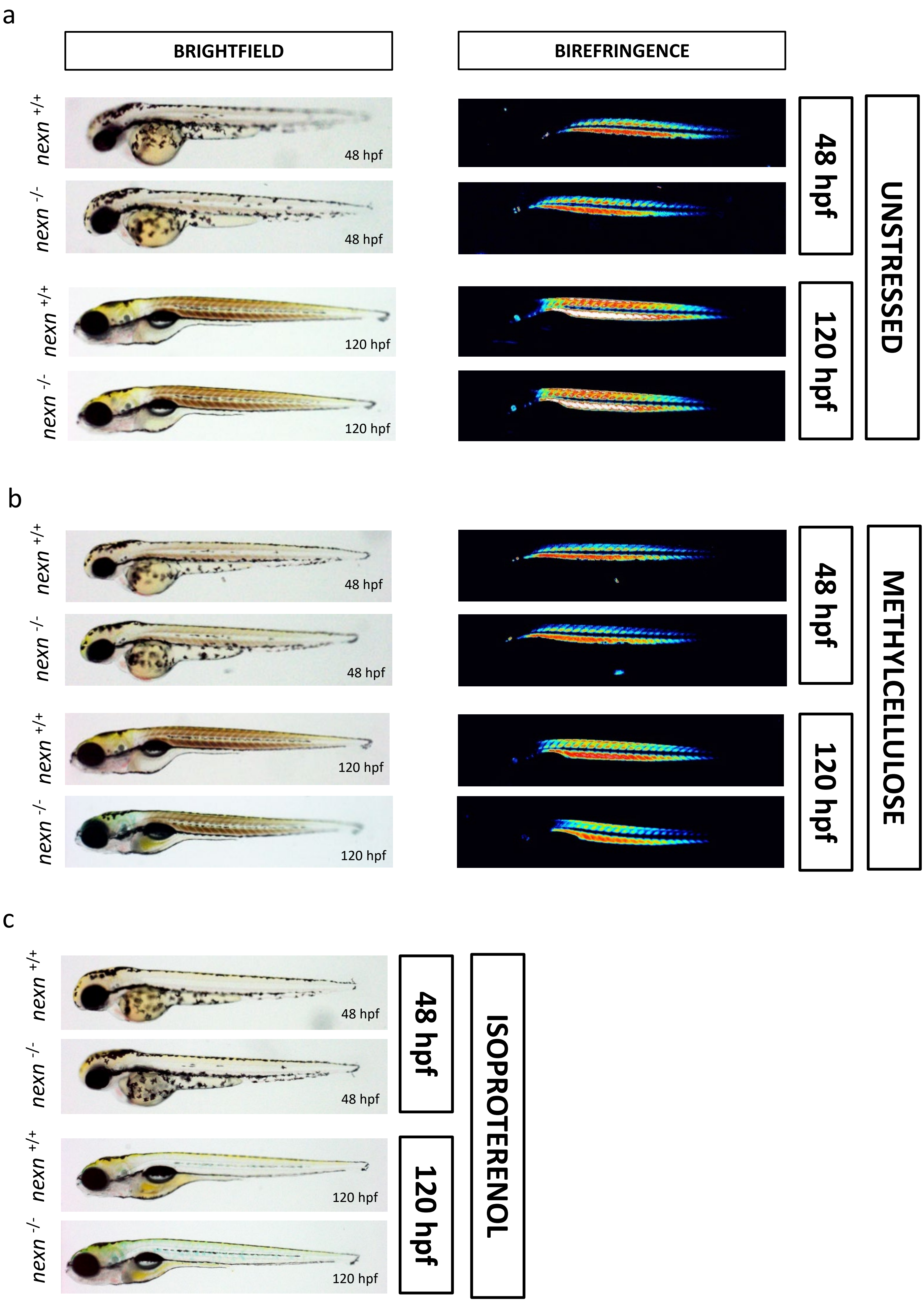

Supplementary Figure S4

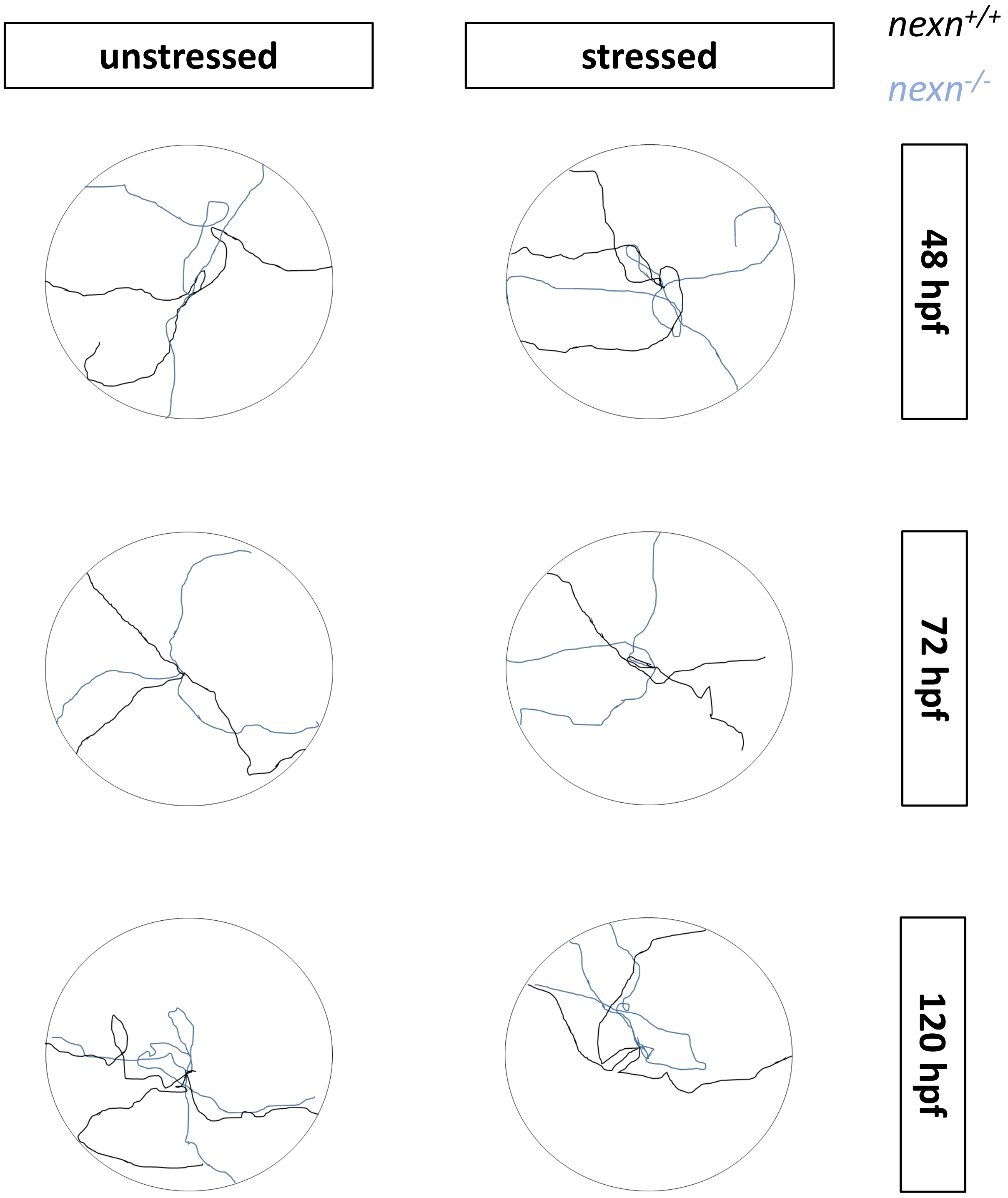

Supplementary Figure S5

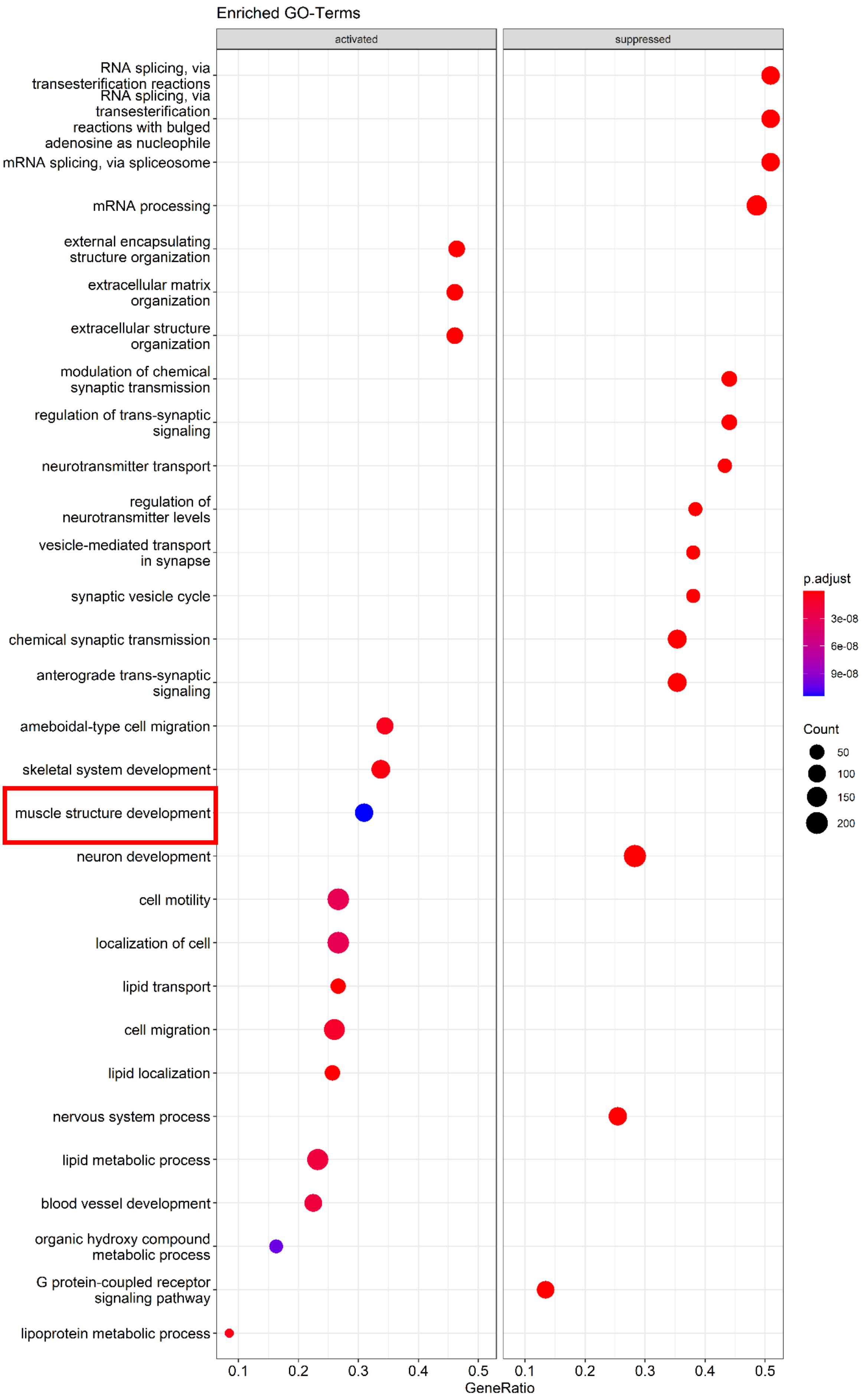

Supplementary Figure S6

	**Sequence (5' – 3')**
uni-tracrRNA [45]	AAACAGCAUAGCAAGUUAAAAUAAGGCUAG UCCGUUAUCAACUUGAAAAAGUGGCACCGAGUCG GUGCU
gRNA_*nexn*_Exon2_antisense	AAACCGCTGTTCATCTCCACCTCT
gRNA_*nexn*_Exon2_sense	TAGGAGAGGTGGAGATGAACAGCG
*nexn*_qRT_fwd	GAAGTGATAGCATGGCGGA
*nexn*_qRT_rev	GTGTTCTCTGAATCAACATCA
*18S*_qRT_fwd	CACTTGTCCCTCTAAGAAGTTGCA
*18S*_qRT_rev	GGTTGATTCCGATAACGAACGA
*rpl13*_qRT_fwd	TCTGGAGGACTGTAAGAGGTATGC
*rpl13*_qRT_rev	AGACGCACAATCTTGAGAGCAG
myhb_qRT_fwd	AAATCTTCCCAATGAATCCACC
myhb_qRT_rev	ACACAACTACAGCATCATACAC
tnni2b.1_qRT_fwd	CGGCAGAGGAAGAAAAACAAA
tnni2b.1_qRT_rev	CATGGCATCAGCAGACATAC
cmlc1_qRT_fwd	GTCACGTCTTGGCAACGCTT
cmlc1_qRT_rev	CAATCTTTGCAGCGTCGGCT
tnnt2c_qRT_fwd	CCAAATTGCGTGCCGAGGAA
tnnt2c_qRT_rev	TGCGACGCTCTAGCAGACAT
tnnt2a_qRT_fwd	TGACATCCACCGTAAGCGCA
tnnt2a_qRT_rev	AACGCTTCTGTCGCTCCCTT
tpm4b_qRT_fwd	TGCGCGAGAAAGCTGAAGGA
tpm4b_qRT_rev	AGTTTCTGAAGCGCAGTGGA
nexn E6 splice MO [8]	ATTGGCAAAGCTGACCTCGCCCATC
Std Ctrl MO	CCTCTTACCTCAGTTACAATTTATA

Supplementary Figure S1: Original immunoblot showing reduced Nexn levels in *nexn*$^{-/-}$ embryos at different exposure times.

Supplementary Figure S2: *nexn* morpholino-knockdown leads to heart and skeletal muscle failure. **(a)** *nexn* MO injected embryos develop (cardio-)myopathy at 72 hpf as indicated in the brightfield and birefringence images. **(b)** Heart rate is at a similar level in *nexn* MO and Std Ctrl MO injected embryos at 72 hpf (N = 3, n = 15, mean ± SD, p = 0.1323 using two-tailed t-test). **(c)** Analysis of ventricular fractional shortening reveals significantly reduced heart contractility in *nexn* MO injected embryos at 72 hpf (N = 3, n = 15, mean ± SD, p < 0.0001 using two-tailed t-test). **(d)** Responsiveness to mechanical stimuli does not differ between *nexn* MO and Std Ctrl MO injected embryos at 72 hpf (N = 3, mean ± SD, p > 0.7000 using Mann-Whitney test). **(e)** Densitometric quantification of birefringence signals shows reduced signal intensity in *nexn* MO injected embryos compared to Std Ctrl MO injected at 72 hpf (N = 3, n = 15, mean ± SD, p < 0.0001 using two-tailed t-test). **(f)** Immunostaining does not reveal differences regarding myofibrillar organization in *nexn* MO injected embryos at 72 hpf. FS: fractional shortening, ns: not significant

Supplementary Figure S3*:* *nexn* knockout does not lead to pericardial edema or blood congestion. Brightfield and birefringence images do not reveal phenotypical differences between *nexn*$^{+/+}$ and *nexn*$^{-/-}$ embryos at 48 and 120 hpf when being **(a)** unstressed, **(b)** stressed with methylcellulose or **(c)** stressed with isoproterenol.

Supplementary Figure S4: Representative illustration of movement pattern during touch-evoked flight response. *nexn*$^{-/-}$ embryos did not have altered motility and showed straight forward flight response after mechanical stimuli (N = 3, n = 15).

Supplementary Figure S5: Dotplot showing the top 15 activated and suppressed Gene Ontology terms. Dots are colored according to their adjusted p-value. Dot size represents counts.

Supplementary Figure S6: Sequences of used oligonucleotides.
